# Dorsal Dislocation of Intermediate Cuneiform With Multiple Cuneiform and Cuboid Fractures Combined With Lisfranc Injury: A Case Report

**DOI:** 10.7759/cureus.50689

**Published:** 2023-12-17

**Authors:** Christos Baltas, Theodoros Mylonas, Dimitrios Lamprou, Alexandros E Koskiniotis, Christina Arnaoutoglou

**Affiliations:** 1 Department of Orthopaedics and Traumatology, General University Hospital of Larissa, Larissa, GRC; 2 Department of Orthopaedics and Traumatology, General University Hospital Of Larissa, Larissa, GRC

**Keywords:** tarsal fractures, cuboid fracture, cuneiform dislocation, lisfranc injury, tarsal bones

## Abstract

Multiple cuneiform fractures combined with isolated intermediate cuneiform dorsal dislocation and cuboid fracture, with disruption of the Lisfranc ligament, are rare injuries. In this study, we present a polytrauma patient who sustained these injuries, his treatment course, and the follow-up period. The patient was operated on the day of the injury and six months after that the results are very satisfactory.

## Introduction

Polytrauma patients with painful and swollen feet should raise our suspicion for midfoot and forefoot fractures or dislocations. Lisfranc injuries are rare injuries that often co-exist with tarsal or metatarsal fractures [1]. Cuboid fractures are uncommon injuries that most often occur with Chopart, Lisfranc, or complex midfoot injuries [2]. Cuneiform fractures and dislocations are also uncommon injury patterns with a few cases reported in the literature [3-10]. In this study, we present a polytrauma patient with a cuboid fracture, multiple cuneiform fractures, and a dorsal dislocation of the intermediate cuneiform. The patient was informed and gave his consent before the submission of the study.

## Case presentation

A 51-year-old male was admitted to the emergency department after having been run over by an agriculture vehicle. He was fully conscious and complained of severe pain in his right hip region and left foot while he had mild pain in his thorax. The initial clinical examination revealed excessive swelling and tenderness on the midfoot, internal rotation, adduction and shortening of his right lower extremity, and some abrasions on his chest wall. He also had ecchymosis an open wound in his right eyebrow and a nosebleed. There was no neurological deficit.

**Figure 1:**
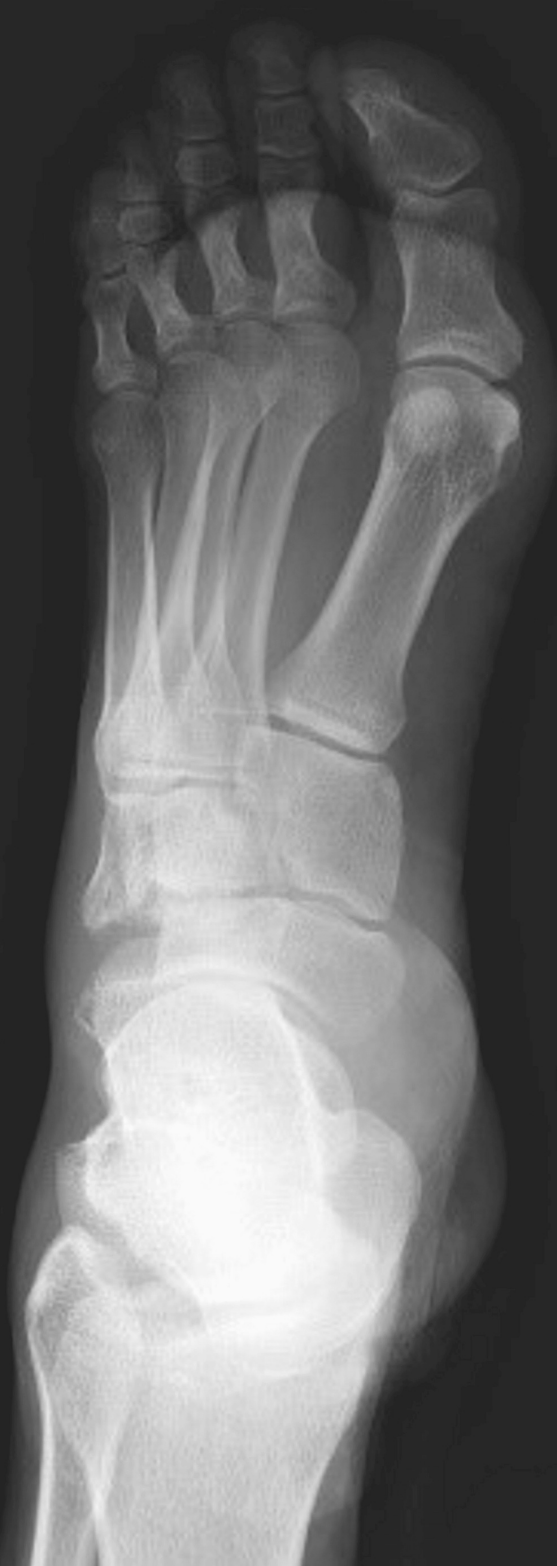
Anteroposterior foot radiograph showing a cuboid fracture

Pelvic x-rays revealed an isolated right hip posterior dislocation. An anteroposterior radiograph of his left foot showed a cuboid fracture but due to a lack of cooperation from the patient, there were no oblique or profile x-rays (Figure 1). Plain radiographs from the thorax revealed no fractures or hemo-pneumothorax. A full-body computed tomography (CT) scan was ordered for further evaluation of the injuries due to the severity of the accident.

**Figure 2:**
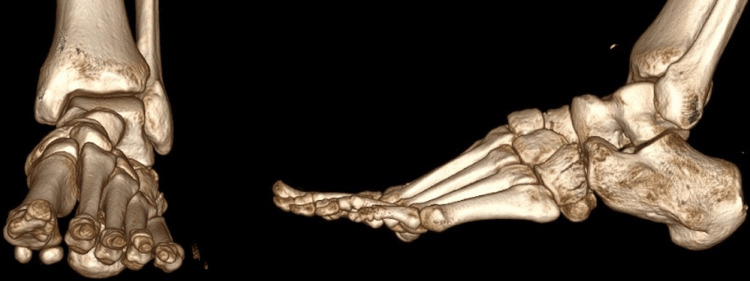
3D foot CT showing dorsal dislocation of the intermediate cuneiform

**Figure 3:**
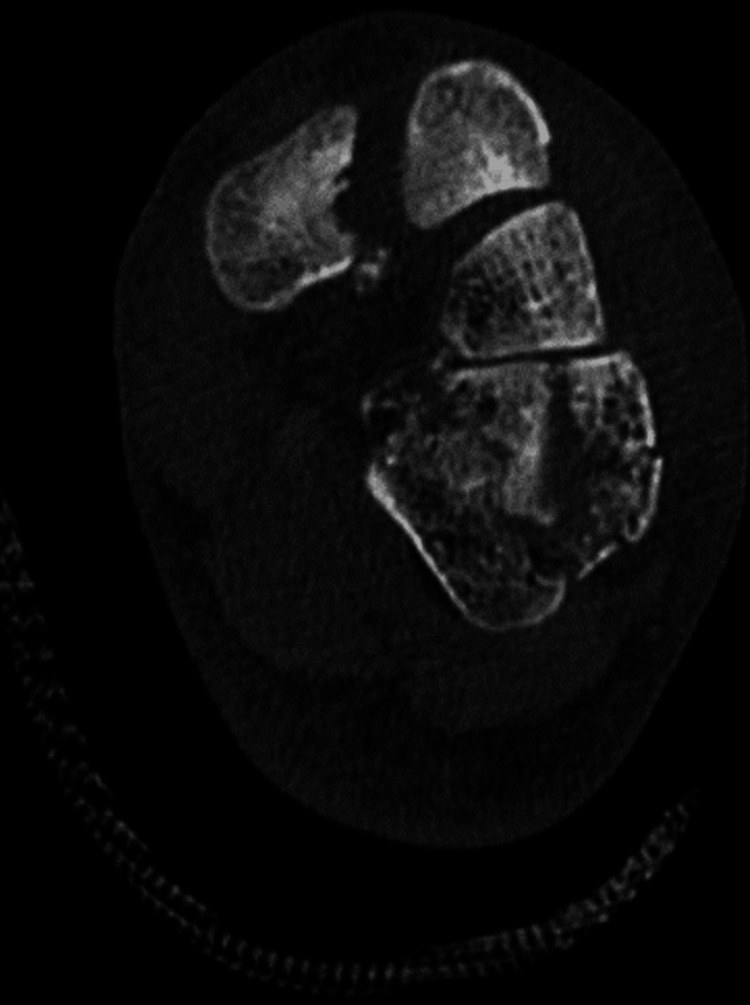
CT scan showing cuboid and cuneiform fractures

CT of the thorax showed no injuries. There was also a right zygomatic fracture, nasal fracture, and a right posterior hip dislocation with small bone fragments in the hip joint. Left foot CT revealed a comminuted cuboid fracture but with adequate axial, sagittal, and coronal volume and well-preserved lateral column length and articular surfaces, dorsal dislocation of the intermediate cuneiform combined with small plantar fractures of all the cuneiforms (Figures 2, 3).

**Figure 4:**
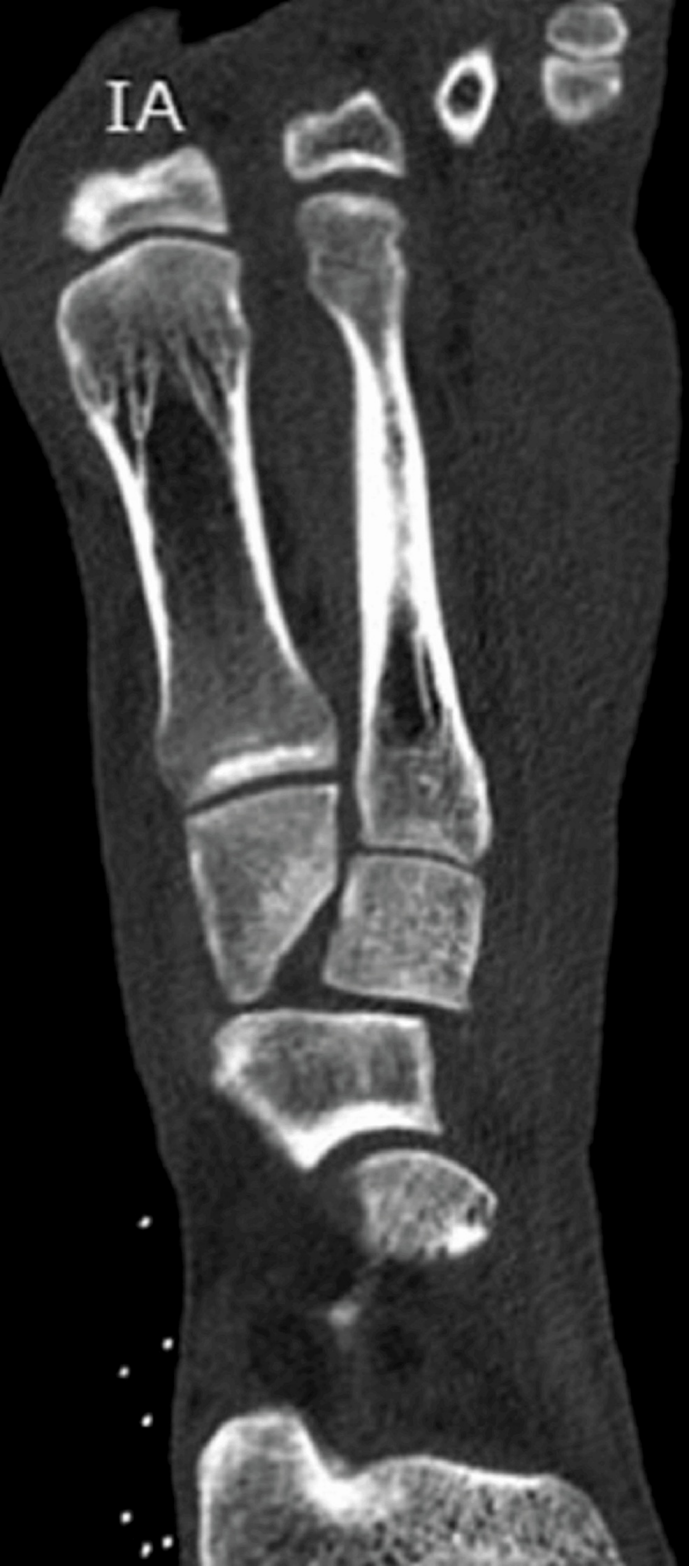
CT that shows Lisfranc joint without damage

The Lisfranc ligament appeared with no damage on the CT, as there was no diastasis between the medial cuneiform and the base of the second metatarsal (Figure 4).

**Figure 5:**
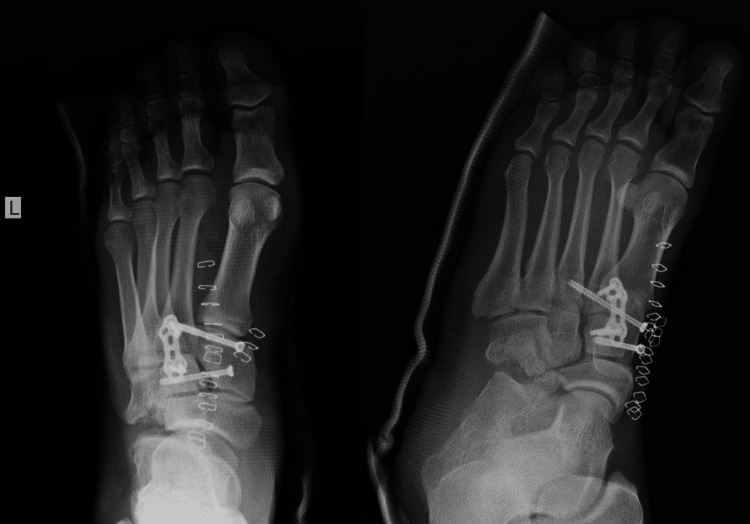
Postoperative foot radiographs

The patient was transferred to the operation room the same day to address the hip dislocation and the foot injury. Through an anteromedial approach of the midfoot, open reduction and internal fixation of the dislocated cuneiform was performed (Figure 5). Intraoperatively was noted that the Lisfranc ligament was ruptured so it had to be fixed. In the first postoperative three weeks, a short leg posterior splint was applied with no weightbearing. After that, the splint was removed, and passive and active range of motion of the ankle was allowed.

**Figure 6:**
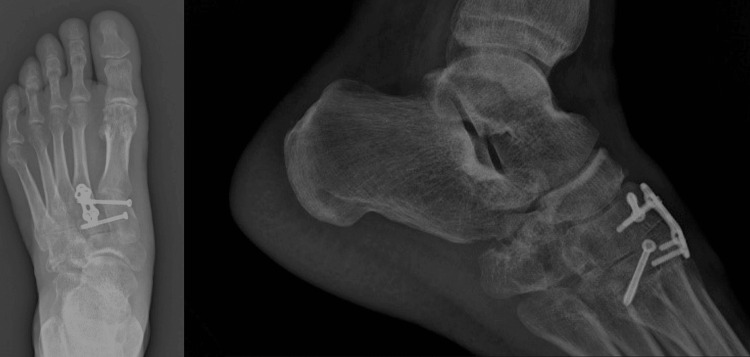
Foot radiographs three months postoperatively

The third week after the injury a hip arthroscopy was performed in order to take off the fragments from the joint. It was a decision taken along with the patient not to proceed with the arthroscopy at the same time as the foot procedure, but to reevaluate his condition with a new CT of the hip. Progressive weight bearing started after six weeks. At three months postoperatively, x-rays revealed no redislocation of the intermediate cuneiform (Figure 6). The patient has a painless foot range of motion and walks with no discomfort at six months follow-up.

## Discussion

The midfoot and forefoot form together a complex anatomic structure. It is divided in three columns. The medial column comprised of the first metatarsal bone and medial cuneiform is the stiffest, whereas the lateral, comprised of the cuboid and fourth and fifth metatarsals is the most mobile column. That complex structure of the foot is rigid due to the function of both the interdigitating joints and the solid ligaments that connect the metatarsals with the tarsal bones. An understanding of the anatomy across the tarsometatarsal joints is essential for the assessment of those injuries. The second metatarsal base is located in a place created by the three cuneiforms. No ligament is present between the first and second metatarsal bases, but the plantar ligament between the second metatarsal base and medial cuneiform is short and thick. In the coronal plane, the base of the second metatarsal creates the apex or keystone in this arch.

Midtarsal injuries vary from minor ligament and soft tissue damage to serious combinations of injuries such as fractures and dislocations that can even put the survival of the foot in jeopardy. The mechanism of injury also varies, from simple twisting injuries to severe crushing forces that produce soft tissue and bone damage. Isolated injuries of the cuneiform or cuboid bones are considered hard to occur, due to the protective anatomy of the region. Fractures in this area should be managed as “combination” fractures or fracture-subluxations until proven otherwise [11].

Clark and Quint described for the first time the isolated intermediate cuneiform dislocation back in 1933 [12]. Until now several case reports have been published with dorsal or plantar intermediate cuneiform dislocations [3-9] combined or not with Lisfranc injuries. Our case study combines those injuries with a fracture of the cuboid bone.

Cuboid fracture is usually a sign of complex and high-energy injuries of the foot. The management of these injuries is related to the particular fracture characteristics. If shortening of the lateral column > 3 mm or articular displacement > 1 mm is to be found, operative intervention is mandatory aiming to avoid unpleasant biomechanical and functional consequences for the foot and various adverse effects such as arthritis in the mid and long-term and stiffness as well as painful gait [13]. In our case, there was no shortening of the lateral column or articular displacement and that is why nonsurgical treatment was chosen for this fracture.

Our management comes in agreement with the qualitative analysis of case studies published by Mabry et al. that support that the nonoperative treatment option of a cuboid fracture has satisfactory outcomes under circumstances [14]. It is important that the patient had also a Lisfranc injury, which was not obvious from either x-rays or CT, and it was confirmed in the operating room. Lisfranc injuries include a spectrum of injuries ranging from pure ligamentous injuries of the Lisfranc joint to displaced and unstable fractures of the midfoot [15]. In a polytrauma patient, the presence of edema and tenderness on the midfoot has always raised suspicion for this type of injury even if the initial radiographic workup is clear.

## Conclusions

The diagnostic approach of a polytrauma patient is often challenging, even though every physician is in theory aware of it. In our case, we had to deal with a patient who sustained a variety of injuries, with the most interesting and challenging among them being the combination of dorsal intermediate cuneiform dislocation with multiple cuneiform and cuboid fractures along with Lisfranc injury. By thoroughly searching the literature we concluded that the cuneiform dislocation, in particular, is a rare medical entity and not many surgeons are experienced in its management. As for the Lisfranc injury, it is usually diagnosed with the assistance of a CT scan, but in our example, no clear signs were apparent, so evaluating it intraoperatively due to high clinical suspicion is mandatory. Finally, the combination of those injuries is something that we were unable to detect in the literature, but every orthopedic surgeon should be aware of its possibility, and this is the reason why we describe it in our study.
